# Evaluation of guide wire cannulation in reduced risk of post - ERCP pancreatitis and facilitated bile duct cannulation 

**Published:** 2012

**Authors:** Shahriar Savadkoohi, Javad Shokri, Hesam Savadkoohi

**Affiliations:** 1 Department of Internal Medicine, Shahid Beheshti Hospital, Babol University of Medical Sciences, Babol, Iran.; 2 Department of Internal Medicine, Islamic Azad University of Tehran, Tehran, Iran.

**Keywords:** ERCP, Pancreatitis, Standard, Guide wire

## Abstract

**Background:**

Pancreatitis is most common complication of post-ERCP and needs to admission at least for one day. The purpose of this study was to assess the efficacy of guide wire for better common bile duct (CBD) cannulation for reducing post-ERCP pancreatitis.

**Methods:**

From April 2010 through March 2011, the patients who needed ERCP and referred to Shahid Beheshti and Rouhani Teaching Hospital were entered into the study. Guide wire cannulation (65 subjects) as case group and 78 cases with standard cannulation as control group were performed on them randomly. Data from these cases were collected and analyzed.

**Results:**

One hundred eighteen (82.5%) patients were females and 28 (17.5%) were males. The mean age of these patients was 56.5±16.8 years. Post- ERCP pancreatitis rate in guide wire group was 6 (9.2%) and in the standard group was 12 (15.4%) (p=0.269). Successful cannulation in these two groups was 67.7% and 67.9%, respectively (p=0.974).

**Conclusion:**

The results show that post- ERCP pancreatitis rate in both groups are similar. Other studies with large number of cases are required to confirm our results.

Acute pancreatitis is the most common complication of post – ERCP and occurred in the expected rate of ERCP pancreatitis ranges from 1%–7% to as high as 12%–31% (1, 2). Post- ERCP pancreatitis is defined as abdominal pain with elevated amylase levels of more than three fold of the upper limit of normal following ERCP and they need admission at least for one night (1). When the abdominal pain is not significant or amylase level is less than three fold, pancreatitis is not considered. Post ERCP – pancreatitis is divided to three stages. In the mild form abdominal pain with hyper- amylasemia of more than three times of normal levels is seen and needs admission of less than 3 days. In the moderate form pancreatitis needs to admission of 4-10 days and in the severe form pancreatitis that need to be admitted for more than 10 days and associated with pseudocyst or needs to intervention as percutaneous drainage or surgery (3). 

Many factors may predispose to post- ERCP pancreatitis, such as sphincter of Oddi dysfunction (SOD), young age, normal bilirubin, history of post- ERCP pancreatitis, difficult cannulation, injection to pancreatic duct, pancreatic sphinctrotomy, biliary balloon dilation, precut sphinctrotomy, female gender, acinarization, no CBD stone, low ERCP cases (3). Probable factors cause post- ERCP pancreatitis includes biliary sphinctrotomy, manometry, and normal CBD diameter (4, 5). Several studies were done to find out modality to reduce post ERCP-pancreatitis. Non-steroid anti inflammatory drugs (NSAIDs), octreotide and pancreatitis duct stent can reduce post- ERCP pancreatitis (6, 7). Recently, CBD cannulation guided by wire was recommended from some extents (2, 8-10). 

This study was designed to evaluate using CBD cannulation with guide wire for reduction of post ERCP- pancreatitis.

## Methods

From April 2010 through April 2011, the patients who need ERCP and referred to Shahid Beheshti and Rouhani Teaching Hospital were entered into the study. These patients were randomly divided into two groups. In the first group (78 cases), and in the second group 65 cases were underwent standard cannulation and guide wire cannulation, respectively. Before performing these procedures, abdominal sonography, and liver function tests were done for all cases and in selected cases MRCP and CT scan were done for them. 

The patients with no successful cannulation or sphinctrotomy were excluded from this study. This study was approved by the internal medicine group and the local Ethics Committee approved this study. These procedures were explained to all the patients. All cases gave their informed consent. After the procedure, all the patients stayed in the hospital overnight. Those who had abdominal pain, liver function test and serum amylase were assessed. The data were collected and analyzed. T-test was used to compare the rate of pancreatitis in these two groups as well as successful cannulation rates.

## Results

One hundred eighteen (82.5%) cases were females and 25 (17.5%) cases were males with mean age of 56.5±16.8 years (ranged 19 to 84 years). The mean age of these two groups were similar (p=0.547) (table 1). Icter was seen in 46.2% cases in guide wire group and in 75% patients in standard cannulation group (p=0.001). 

Successful cannulation was seen in 67.7% of the guided wire group and in 67.9% of the standard cannulation group (p=0.974). Standard sphynctrotomy was 67.7% and 67.9% in guide wire and standard cunnulation group (p=0.97). The other characteristics of the patients in both groups are shown in table 1. Hyperamylasemia was seen in 49.2% cases in guide wire group and in 42.9% in standard cunnulation group (p=0.6). The patients with post ERCP-pancreatitis in guide wire group and in control group were seen in 6 (9.2%) and in 12 (%15.4%) patients (p=0.269).

**Table1:** Characteristics and ERCP results in these two group

pvalue	SC	GW	Group Variable
0.290	63 (80.8) 15 (19.2)	55 (84.6) 10 (15.4)	Sex Female Male
0.290	54.79±18.84	57.78±13.87	Age (mean±SD)
0.001	59 (75.6)	30 (46.2)	Icter
0.974	53 (67.9)	44 (67.7)	Successful deep cannulation
0.974	53 (67.9)	44 (67.7)	Standard sphinctrotomy
0.974	25 (32.1)	21 (32.3)	Precut
0.603	35 (44.9)	32 (49.2)	Hyperamylasemia
0. 269	12 (15.4)	6 (9.2)	Pancreatitis

GW, guide wire

SC, standard cunnulation

## Discussion

In this study, we found that overall post- ERCP pancreatitis rate was 18 (12.6%). The rate of post- ERCP pancreatitis in wire guided group was 9.2% and in standard cunnulation group was 15.4%. We found no statistical difference regarding development of post- ERCP pancreatitis in both groups. Our findings were similar to the report of Everson et al. on 300 cases in Brazil (9). The rates of pancreatitis in their study in wire guided group were 8.6% and in standard cunnulation was 16.6%. Pancreatitis in their study was mild compared to 3 of our cases who had moderate to severe pancreatitis. Other studies also showed no significant differences regarding post-ERCP pancreatitis both in metaanalysis and researched studies (8, 11, 12). But in an Italian meta-analysis study showed that the wire-guided technique (83.3%) increased the primary cannulation rate and reduced the risk of post-ERCP pancreatitis compared with the standard contrast-injection method (74.9%) (12). In a study in Greece on 217 patients who underwent ERCP with wire guided cannulation and standard cannulation showed that post ERCP pancreatitis significantly was lower in wire guided cannulation (13). Several other studies which compared post-ERCP pancreatitis following guide wire cannulation versus standard cannulation showed no differences for the development of post-ERCP pancreatitis (2, 6, 9, 14, 15). 

Even experts believe that the use of a sphincterotome with guide wire increases the success rate of selective bile duct cannulation in cases that this has not been accomplished with a standard catheter (16). Park et al. believe that the effect of ERCP depends on high success rates and low complication rates (17). Despite several randomized, controlled trials and metaanalyses that showed a WGC can prevent post-ERCP pancreatitis, conflicting data still exist (18, 19). 

This discrepancy in our study may be due to several factors such as low number of our cases, early pre-cut compare with late pre-cut which were not determine.

In conclusion, the results of our study show that post ERCP –pancreatitis rate in both groups are similar. Other studies with large number of cases are required to confirm our results.
